# Sentinel nodes identified by computed tomography-lymphography accurately stage the axilla in patients with breast cancer

**DOI:** 10.1186/1471-2342-13-42

**Published:** 2013-12-09

**Authors:** Kazuyoshi Motomura, Hiroshi Sumino, Atsushi Noguchi, Takashi Horinouchi, Katsuyuki Nakanishi

**Affiliations:** 1 Departments of Surgery, Osaka Medical Center for Cancer and Cardiovascular Diseases, 1-3-3 Nakamichi, Higashinari-ku 537-8511Osaka, Japan; 2 Departments of Radiology, Osaka Medical Center for Cancer and Cardiovascular Diseases, 1-3-3 Nakamichi, Higashinari-ku 537-8511Osaka, Japan

**Keywords:** Sentinel node, Breast cancer, Computed tomography, Lymphography, Staging

## Abstract

**Background:**

Sentinel node biopsy often results in the identification and removal of multiple nodes as sentinel nodes, although most of these nodes could be non-sentinel nodes. This study investigated whether computed tomography-lymphography (CT-LG) can distinguish sentinel nodes from non-sentinel nodes and whether sentinel nodes identified by CT-LG can accurately stage the axilla in patients with breast cancer.

**Methods:**

This study included 184 patients with breast cancer and clinically negative nodes. Contrast agent was injected interstitially. The location of sentinel nodes was marked on the skin surface using a CT laser light navigator system. Lymph nodes located just under the marks were first removed as sentinel nodes. Then, all dyed nodes or all hot nodes were removed.

**Results:**

The mean number of sentinel nodes identified by CT-LG was significantly lower than that of dyed and/or hot nodes removed (1.1 vs 1.8, p <0.0001). Twenty-three (12.5%) patients had ≥2 sentinel nodes identified by CT-LG removed, whereas 94 (51.1%) of patients had ≥2 dyed and/or hot nodes removed (p <0.0001). Pathological evaluation demonstrated that 47 (25.5%) of 184 patients had metastasis to at least one node. All 47 patients demonstrated metastases to at least one of the sentinel nodes identified by CT-LG.

**Conclusions:**

CT-LG can distinguish sentinel nodes from non-sentinel nodes, and sentinel nodes identified by CT-LG can accurately stage the axilla in patients with breast cancer. Successful identification of sentinel nodes using CT-LG may facilitate image-based diagnosis of metastasis, possibly leading to the omission of sentinel node biopsy.

## Background

Sentinel node biopsy has been established as a standard of care in the treatment of breast cancer. This technique represents a minimally invasive, highly accurate method of axillary staging and is an alternative to conventional axillary lymph node dissection [1-5]. Controversy exists regarding the several technical and clinical aspects of sentinel node biopsy. One of the most important issues is how many and which axillary lymph nodes need to be removed as sentinel nodes for accurate axillary staging. Sentinel node biopsy using dye and/or radioisotopes often results in the identification and removal of multiple nodes as sentinel nodes, although most of these nodes could be non-sentinel nodes because the dye or radioisotope may migrate from sentinel nodes into additional non-sentinel nodes. The excision and examination of multiple sentinel nodes reduces the false negative rate, but removal of a large number of sentinel nodes increases morbidity and is time-consuming [6]. While some researchers have suggested that all dyed and/or radioactive nodes should be removed [7,8], others have proposed that the sentinel node biopsy procedure should be stopped after some lymph nodes or all nodes with radioactive counts greater than 10% of the hottest node have been removed [9-14].

Recently, sentinel nodes have been reported to be well-identified using computed tomography-lymphography (CT-LG) in patients with breast cancer [15-19]. Lymph flow and sentinel nodes were successfully visualized by interstitial injection of CT contrast agent.

This study investigated whether CT-LG can distinguish sentinel nodes from non-sentinel nodes by visualization of the lymphatic channel and whether sentinel nodes identified by CT-LG can accurately stage the axilla in patients with breast cancer.

## Methods

### Patient selection

One hundred and eighty-four consecutive patients with clinical T1-2 breast cancers and clinically negative nodes who underwent sentinel node biopsy at Osaka Medical Center for Cancer and Cardiovascular Diseases between February 2008 and December 2010 were enrolled in this study. Patients with nonpalpable breast cancer, prior axillary surgery or pregnancy were excluded. Patients with a contraindication to CT or a known allergy to the contrast agent were also excluded. The institutional review board of Osaka Medical Center for Cancer and Cardiovascular Diseases approved the study, and written consent was obtained from all patients.

### Sentinel node localization using CT-LG

Interstitial CT-LG was performed using a multidetector row helical CT scanner (Light Speed VCT; GE Healthcare, Milwaukee, WI, USA). Contiguous 1.25-mm-thick CT images from the upper thorax to axillary regions were obtained once before administration of the contrast agent. CT scanning with a detector of 0.625 mm, 64 rows was operated at 120 kV, 300 to 400 Auto-mA, 35 cm field of view, 512 x 512 matrix, section spacing of 1 mm, and table speed of 1.55 mm/0.5 sec.

**Figure 1:**
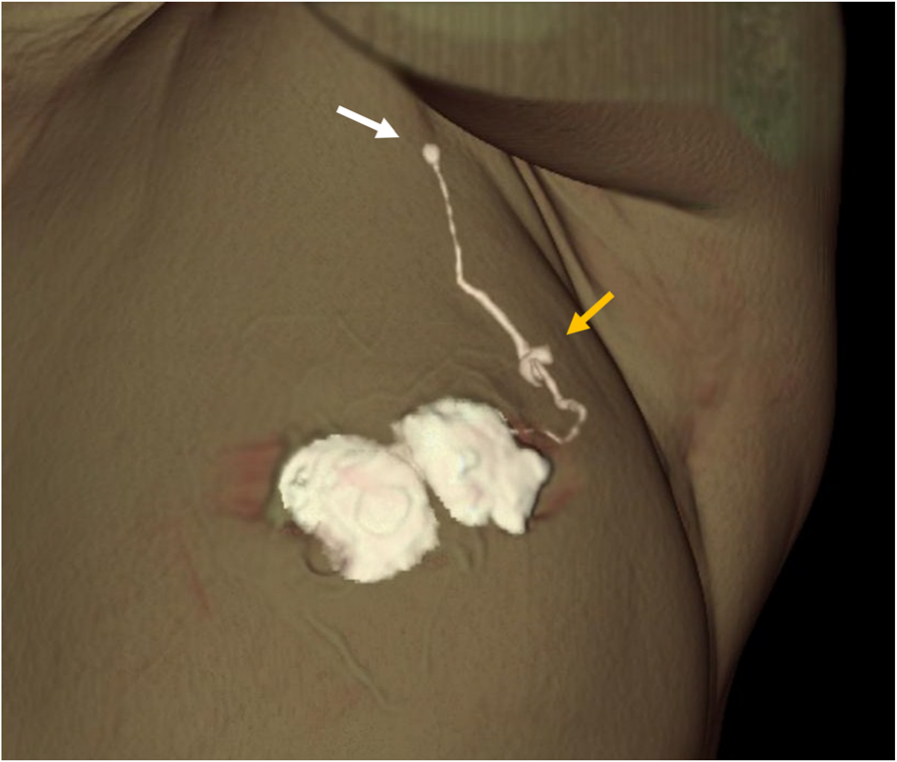
**Three-dimensional computed tomography-lymphography (CT-LG) reconstructed from the first post-contrast images.** Contrast agent was injected intradermally into the skin overlying the breast tumor and the subareolar skin. Lymphatic vessels drained into a single axillary sentinel node (yellow arrow). CT-LG can visualize lymph flow and can distinguish sentinel nodes from non-sentinel nodes (white arrow).

**Figure 2:**
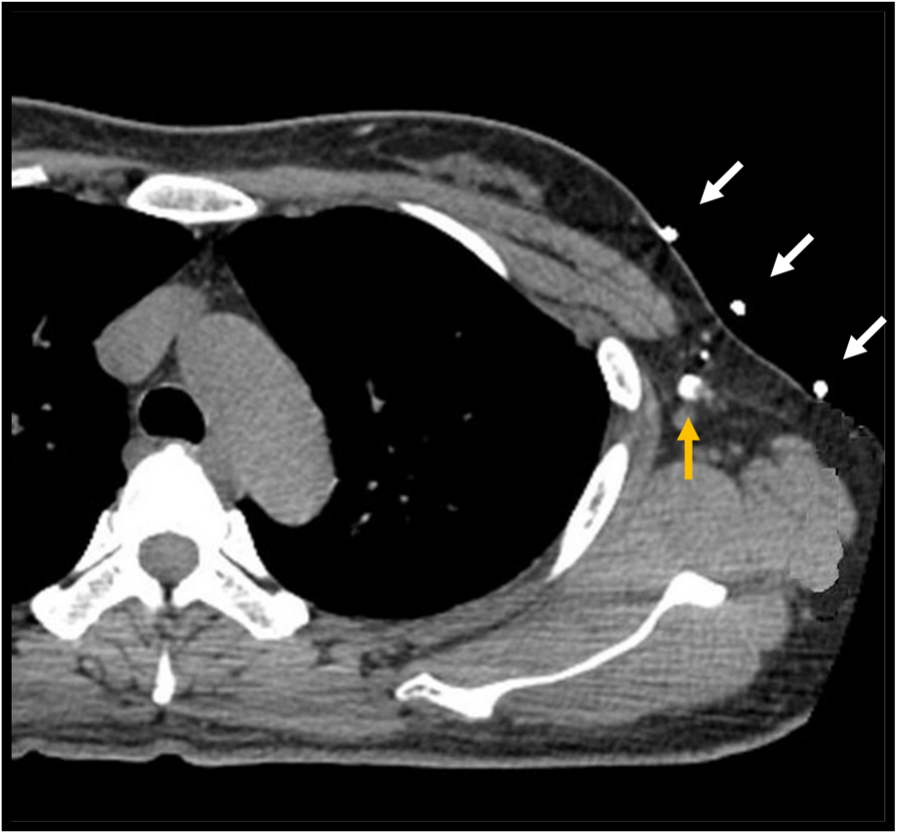
Sentinel node (yellow arrow) identified by axial computed tomography with localizing marker (white arrows).

Transaxial CT images were reconstructed with a 1.25-mm pitch and slice thickness of 0.3 mm. 3D CT images were reconstructed from the post-contrast CT images at each time point with volume-rendering techniques and, if necessary, a workstation (GE Advantage Workstation, version 4.3; GE Healthcare) was used to further examine lymph flow and sentinel nodes (Figure 1). Each patient was placed in the supine position and their arms were elevated. After local anesthesia with subcutaneous injection of 2 ml of 2% procaine hydrochloride, a 6-ml dose of iopamidol (Iopamiron 370; Bayer Schering Pharma, Osaka, Japan) was injected intradermally into the skin overlying the breast tumors and into the subareolar skin. A CT scan was performed after massaging the injection site of iopamidol for one minute. A localizing marker, which is usually used for CT-guided lung nodule biopsy, was attached to the skin at the axilla to identify the sentinel node location over the skin (Figure 2) [20]. Sentinel nodes were identified as the first stained nodes on the lymphatic flow from the injection sites of the contrast agent.

**Figure 3:**
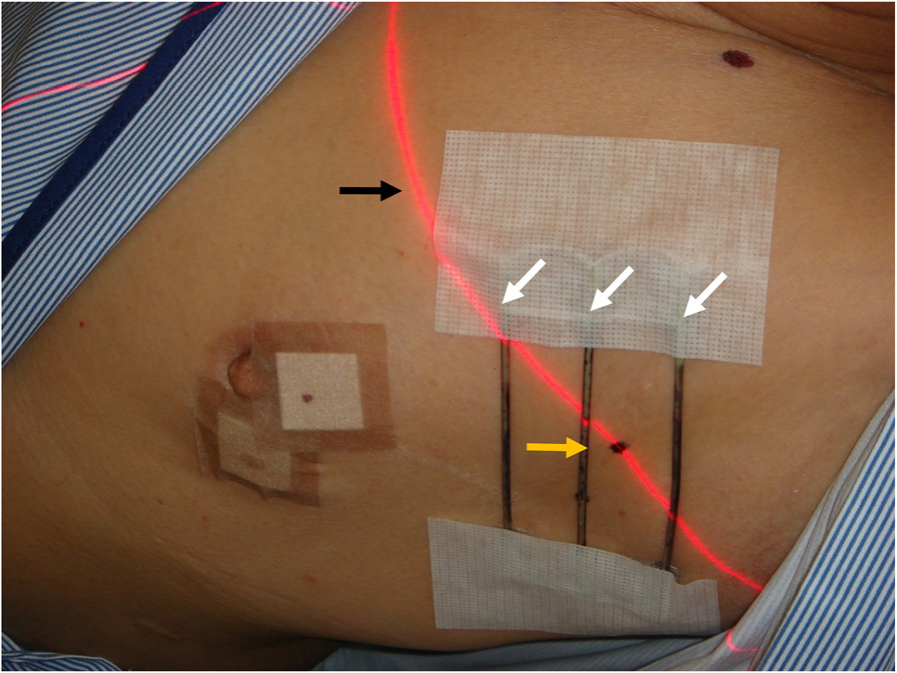
**The sentinel node was indicated precisely by the crossing point of the localizing marker (white arrows) and the computed tomography plane lights (black arrow).** The site was marked on the skin surface using an oil pen (yellow arrow).

The sentinel node location was identified on the CT image and was indicated precisely by the crossing point of the localizing marker and the CT plane lights. The site was marked on the skin surface using an oil pen (Figure 3).

### Surgery

Sentinel node biopsy was performed as described previously [21-23]. In brief, intradermal or intradermal and subareolar injection of 0.3 mL of 37 MBq (1 mCi) Tc-99 m tin colloid the day before surgery and peritumoral or intradermal and subareolar injection of 5 mL indocyanine green (ICG, Diagnogreen 0.5%; Daiichi Pharmaceutical Co. Ltd., Nihonbashi, Tokyo, Japan) 10 minutes before surgery were performed, and then the injection site was massaged manually for one minute. Lymphoscintigraphy was performed 2–3 hours after the radioisotope injection.

Breast surgery was performed before axillary surgery in all patients to minimize the influence of radioactivity from the injection site [21-23]. For surgery, the elevated arm was placed as close as possible in the same position as during CT marking. Hot nodes were identified using a gamma probe (neo2000; Neoprobe Corporation, Dublin, OH, USA). Dyed and/or hot nodes located just under the markers using CT images were defined as sentinel nodes and were removed first. All dyed nodes or all nodes with an ex vivo radioisotope count of twofold or greater than the axillary background were then removed.

### Histopathology

Sentinel nodes and dyed and/or hot nodes were serially sectioned at 2 mm intervals. Hematoxylin and eosin sections of these nodes were prepared from each 2-mm slice. An additional 4-μm section was cut and stained with immunohistochemistry (IHC) using the avidin-biotinylated peroxidase complex technique with the mouse monoclonal antibody against cytokeratin (NCL-CK19; Novocastra Laboratories Ltd., Newcastle, UK or AE1/AE3; Thermoelectron Corp., Waltham, MA, USA). Nodes with isolated tumor cells identified by IHC were considered to be metastasis negative in this study, according to the tumor node metastasis categories defined in the 6th edition of the Union Internacional Contra la Cancrum TNM categories [24].

### Statistical analysis

Fisher’s exact test and paired t-test was used for statistical analysis. Differences were considered to be significant when P <0.05.

## Results

The mean age of the 184 patients was 55.7 (range, 31–79) years old and the mean tumor size was 20.8 (range, 0.2-90) mm. Patient and tumor characteristics are summarized in Table 1. The mean number of sentinel nodes identified by CT-LG was significantly lower than that of dyed and/or hot nodes removed (1.1 vs 1.8, p <0.0001). One hundred sixty-one patients (87.5%) had 1 sentinel node identified by CT-LG removed, 21 (11.4%) had 2 sentinel nodes removed, and 2 patients (1.1%) had 3 sentinel nodes removed. Twenty-three (12.5%) patients had ≥2 sentinel nodes identified by CT-LG removed, whereas 94 (51.1%) of patients had ≥2 dyed and/or hot nodes removed (p <0.0001). Pathologic evaluation demonstrated that 47 (25.5%) of 184 patients had metastasis to at least one node. Three hundred twenty-eight dyed and/or hot nodes were removed, of which 57 (17.4%) had metastatic deposits. Two hundred eleven sentinel nodes were removed, of which 52 (24.6%) had metastatic deposits. Sixteen (30.8%) had micrometastases and 36 (69.2%) had macrometastases. All 47 patients demonstrated metastases to at least one of the sentinel nodes identified by CT-LG. No patient with negative sentinel nodes had metastases in other dyed and/or hot nodes.

**Table 1:** Patient characteristics

	**No. of patients**	**%**
Age, years		
<50	56	30.4
≥50	128	69.6
Tumor size, cm		
≤2	114	62.0
>2, ≤5	65	35.3
>5	5	
Tumor location		
Upper outer	101	54.9
Upper inner	35	19.0
Lower outer	26	14.1
Lower inner	9	4.9
Central	10	5.4
Multicentric	3	1.6
Tumor histology		
Invasive ductal	161	87.5
Invasive lobular	8	4.3
Ductal carcinoma in situ	10	5.4
Others	5	2.7
Type of surgery		
Lumpectomy	178	96.7
Mastectomy	6	3.3
Estrogen receptor		
Positive	150	81.5
Negative	34	18.5
HER-2/neu		
Positive	28	15.2
Negative	156	84.8

CT-LG could visualize lymph flow and accurately identify sentinel nodes in 179 (97.3%) of 184 patients (Figure 1). In 4 of the other 5 patients, only one sentinel node was identified and it was not necessary to distinguish sentinel nodes from non-sentinel nodes by visualizing lymph flow. In one patient, two nodes were identified and they could not be distinguished. No extra-axillary sentinel nodes, such as internal mammary or supraclaviculal sentinel nodes, were identified. Hot spots could be identified over the skin using a gamma probe on all markers of the sentinel node location by CT-LG. There were no adverse events associated with CT-LG.

## Discussion

Sentinel node biopsy using dye and radioisotopes often results in the removal of multiple sentinel nodes. It remains unclear how many and which lymph nodes must be removed as sentinel nodes for accurate axillary staging. Removing only the first node identified or removing only the hottest node may not complete the sentinel node biopsy. Wong et al. reported a false-negative rate of 14.4% if only the first node had been taken [25]. Martin et al. demonstrated that the positive sentinel node was not the most radioactive node in 20% of cases with multiple sentinel nodes, and the false-negative rate was likely to be much higher (12%) if only the most radioactive sentinel node was removed [26]. Some researchers recommend that all lymph nodes above a predefined threshold of the ex vivo count of the hottest sentinel node should be removed. The 10% rule is one of the most common guidelines to define a radioactive sentinel node and dictates removal of all sentinel node with counts >10% of the most radioactive node. Martin et al. demonstrated that the false-negative rate would be 13% if only the hottest node was removed and 5.8% if the 10% rule was applied [13]. Sixty percent of patients had >1 sentinel node removed and the mean number of sentinel nodes per patient was 1.96. Chung et al. reported that only 1.7% of all sentinel node-positive patients had positive sentinel nodes with counts <10% radioactive counts of the hottest node [14]. Sixty-five percent of patients had >1 sentinel node removed. More than one sentinel node needs to be removed in many patients according to the 10% rule. Others recommended that the procedure can be stopped after a certain number of lymph nodes have been removed. Zervos et al. found 98% of positive sentinel nodes were found in the first three nodes removed [9]. McCarter et al. reported that 98% of positive sentinel nodes were found when the first three nodes were removed [7]. Shrenk et al. found 99% of positive sentinel nodes within the first two nodes and 100% of positive nodes within the first three nodes [8]; however, they both concluded that all dyed and/or hot nodes should be removed to decrease the false-negative rate. Chagper et al. found that only 89.7% of positive sentinel nodes was identified within the first three nodes and did not recommend removing only three sentinel nodes because of a high false-negative rate of 10.3% [27]. Zakaria et al. demonstrated that 98% of patients with positive nodes were found by the third sentinel node, and 100% were found by the fourth sentinel node [10]. Yi et al. demonstrated that >99% of positive sentinel nodes were identified in one of the first five lymph nodes removed [11]. Woznick et al. reported that all positive sentinel nodes were identified within the first six nodes removed [12]. Removal of many more nodes was associated with a lower false-negative rate, but could worsen the morbidity of the sentinel node biopsy. Wilke et al. demonstrated an increased incidence of axillary seroma and wound infection when more than four sentinel nodes were removed [6].

**Figure 4:**
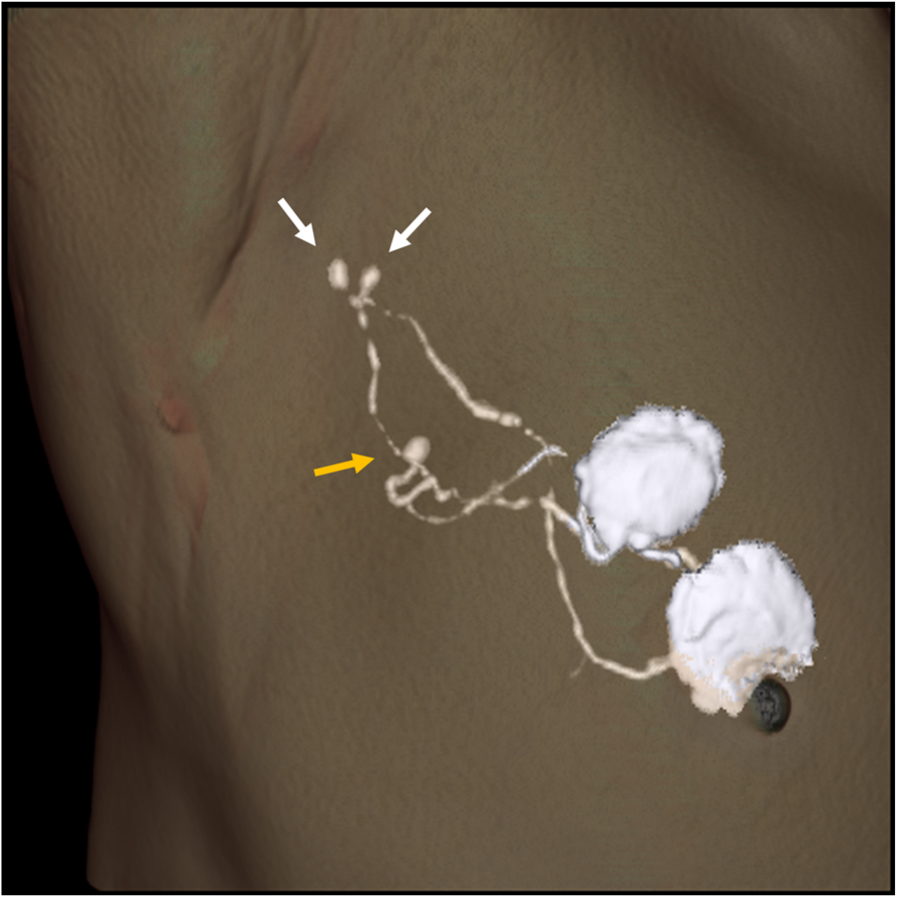
**Three sentinel nodes were identified by computed tomography -lymphography (CT-LG).** One (yellow arrow) might have been missed when sentinel node biopsy was performed without CT-LG because it was located far from the other two nodes (white arrows) and was influenced by radioactivity from the injection site.

In the present study, we demonstrated that sentinel nodes could be successfully identified in 183 of 184 patients and metastases could be detected in all 47 patients with positive nodes. CT-LG could visualize lymph flow and accurately distinguished sentinel nodes from dyed and/or hot non-sentinel nodes (Figure 1). Overall, 87.5% of patients had only one sentinel node removed; 12.5% of patients required removal of ≥2 sentinel nodes, whereas 51.1% of patients required removal of ≥2 dyed and/or nodes, which was statistically significant (p <0.0001). Increased operative time, procedure and pathology cost, and complication rate are associated with the removal of larger numbers of sentinel nodes [6]. It is advantageous to remove only one lymph node as a sentinel node if these nodes do not reduce mapping accuracy. Although four CT studies were performed per patient in this study to clarify which CT studies were really required, a single post-contrast CT scan may be sufficient because the first contrast CT image was able to identify lymphatic channels and sentinel nodes accurately. A further study is required to confirm the hypothesis. Another advantage of CT-LG is that we can identify how many and which node should be removed as sentinel nodes preoperatively. Moreover, we can also identify the location of sentinel nodes, including the depth from the skin, and in surrounding organs such as the chest wall, muscle and vessels according to the appearance of the axial CT and 3D-CT images (Figures 1 and 2). Some multiple sentinel nodes, which may be missed when sentinel node biopsy is performed using dye and/or radioisotopes without CT-LG because they are located far from other nodes and influenced by radioactivity from the injection site, could be accurately identified using CT-LG (Figure 4). Furthermore, axial CT and 3D-CT images enabled the demonstration of the shapes and sizes of sentinel nodes (Figures 1 and 4). The use of 3D images is useful for identification and removal during sentinel node biopsy.

On the other hand, our study had a few limitations, including that the false negative rate of this procedure for axillary staging could not be shown because axillary lymph node dissection was not performed. Sentinel node biopsy has now become the standard of care and it is impracticable to perform axillary lymph node dissection after sentinel node biopsy for sentinel node-negative patients, even in the trial setting. However, all 47 patients with positively dyed and/or hot nodes demonstrated metastases to at least one of the sentinel nodes identified by CT-LG and no patient with negative sentinel nodes had metastases in other dyed and/or hot nodes; therefore, the diagnostic accuracy of sentinel nodes for axillary staging is similar to that of sentinel nodes identified by dye and radioisotopes, demonstrated in our previous study with 100% sensitivity, 100% specificity, and 100% accuracy [21]. Intradermal injection of enhanced agents cannot identify extra-axillary sentinel nodes, but removal of sentinel nodes in such a region has not been performed recently. Chagper et al. demonstrated that axillary sentinel nodes are usually identified even when lymphoscintigraphy shows drainage to the internal mammary nodes alone [28].

Lymphoscintigraphy can sometimes show several hot nodes and lymphatic flow before sentinel node biopsy [29]; however, it is not easy to clearly distinguish sentinel nodes from non-sentinel nodes in many cases because of unclear lymph flow images and only identification of the hottest node in spite of the existence of more than one hot node. CT-LG can identify sentinel nodes clearly. Moreover, if sentinel nodes accurately be diagnosed using imaging, even sentinel node biopsy can be omitted. CT itself is reported to be insufficient to evaluate the presence of metastases in sentinel nodes [30]. We recently tried to perform MR imaging with superparamagnetic iron oxide enhancement for the accurate detection of metastases in sentinel nodes localized by CT-LG in patients with breast cancer [31]. The sensitivity, specificity, and accuracy of MR imaging for the diagnosis of sentinel node metastases were 84.0%, 90.9%, and 89.2%, respectively. In 4 of 10 patients with micrometastases, metastases were not detected, but all 15 patients with macrometastases were successfully identified. This promising procedure may avoid even sentinel node biopsy when the sentinel node is diagnosed as disease-free on MR imaging.

## Conclusions

CT-LG could distinguish sentinel nodes from non-sentinel nodes, and sentinel nodes accurately staged the axilla in patients with breast cancer. Applying this procedure may end the dispute regarding how many and which axillary lymph nodes need to be removed as sentinel nodes for accurate axillary staging. Successful identification of sentinel nodes using CT-LG may facilitate image-based diagnosis of metastasis, possibly leading to the omission of sentinel node biopsy.

## Abbreviations

CT: Computed tomography; MR: Magnetic resonance; CT-LG: Computed tomography-lymphography; ICG: Indocyanine green; IHC: Immunohistochemistry.

## Competing interests

The authors declare that they have no competing interests.

## Authors’ contributions

KM contributed to the conception and design of the study, data analysis and drafted the manuscript. HS contributed the analysis and interpretation of the data of CT. AN provided methodological advice. TH and KN contributed to the conception and design of the study, analysis and interpretation of the data. All authors read and approved the final manuscript.

## Pre-publication history

The pre-publication history for this paper can be accessed here:

http://www.biomedcentral.com/1471-2342/13/42/prepub
